# PHLDA3 impedes somatic cell reprogramming by activating Akt-GSK3β pathway

**DOI:** 10.1038/s41598-017-02982-9

**Published:** 2017-06-06

**Authors:** Mengran Qiao, Mian Wu, Ronghua Shi, Wanglai Hu

**Affiliations:** 1 CAS Key Laboratory of Innate Immunity and Chronic Disease, CAS Center for Excellence in Cell and Molecular Biology, Innovation Center for Cell Signaling Network, School of Life Sciences, University of Science & Technology of China, Hefei, 230027 China; 2 Department of Immunology, Anhui Medical University, Hefei, 230032 China

## Abstract

Reprogramming of adult somatic cells into induced pluripotent stem cells holds great promise in clinical therapy. Increasing evidences have shown that p53 and its target genes play important roles in somatic cell reprogramming. In this study, we report that PHLDA3, a p53 target gene, functions as a blockage of iPSCs generation by activating the Akt-GSK3β pathway. Furthermore, PHLDA3 is found to be transcriptionally regulated by Oct4. These findings reveal that PHLDA3 acts as a new member of the regulatory network of somatic cell reprogramming.

## Introduction

Direct reprogramming of differentiated somatic cells by defined transcription factors, such as Oct4 (O), Sox2 (S), Klf4 (K), and c-Myc (M), can generate induced pluripotent stem cells (iPSCs) exhibiting embryonic stem cell (ESC)-like characteristics^1–5^. Both ESCs and iPSCs depend on the transcription networks that are governed by stem cell-specific transcription factors^6^. While the maintenance of self-renewal is mainly regulated by LIF-STAT3^7, 8^ and BMP pathways^9^, the differentiation of ESCs is known to be promoted by MEK-ERK and GSK3β signaling^10^. The downstream transcription factors Oct-4, Sox2 and Nanog positively regulate transcription of all pluripotency circuitry to activate genes that sustain the undifferentiated state and suppress genes that promote differentiation^11–13^.

Pleckstrin homology-like domain family A, member 3 (PHLDA3) is a p53-regulated repressor of Akt. It is a direct p53 target^14^. It contains a PH domain that competes with the PH domain of Akt for binding to membrane lipids, thereby inhibiting Akt translocation to the cellular membrane and its activation. PHLDA3 gene is also a tumor suppressor, inactivation of which can lead to the development of PanNETs (Pancreatic neuroendocrine tumors)^15^. PHLDA3-deficient mice frequently develop islet hyperplasia as a result of enhanced islet cell proliferation and an increase in islet cell size^15^.

Most of the previous studies focusing on PHLDA3 are tumorigenesis-related cell behaviors. Its function in somatic reprogramming and stem cell maintaining has not yet been reported. Here, we report that PHLDA3 impedes the generation of iPS cells. Mechanistically, PHLDA3 activates the Akt-GSK3β pathway during the reprogramming process. Also, PHLDA3 is transcriptionally regulated by Oct4. These findings reveal that PHLDA3 acts as a new member of the regulating network during somatic cell reprogramming.

## Results

### PHLDA3 expression decreases during the reprogramming of iPS cells

**Figure 1:**
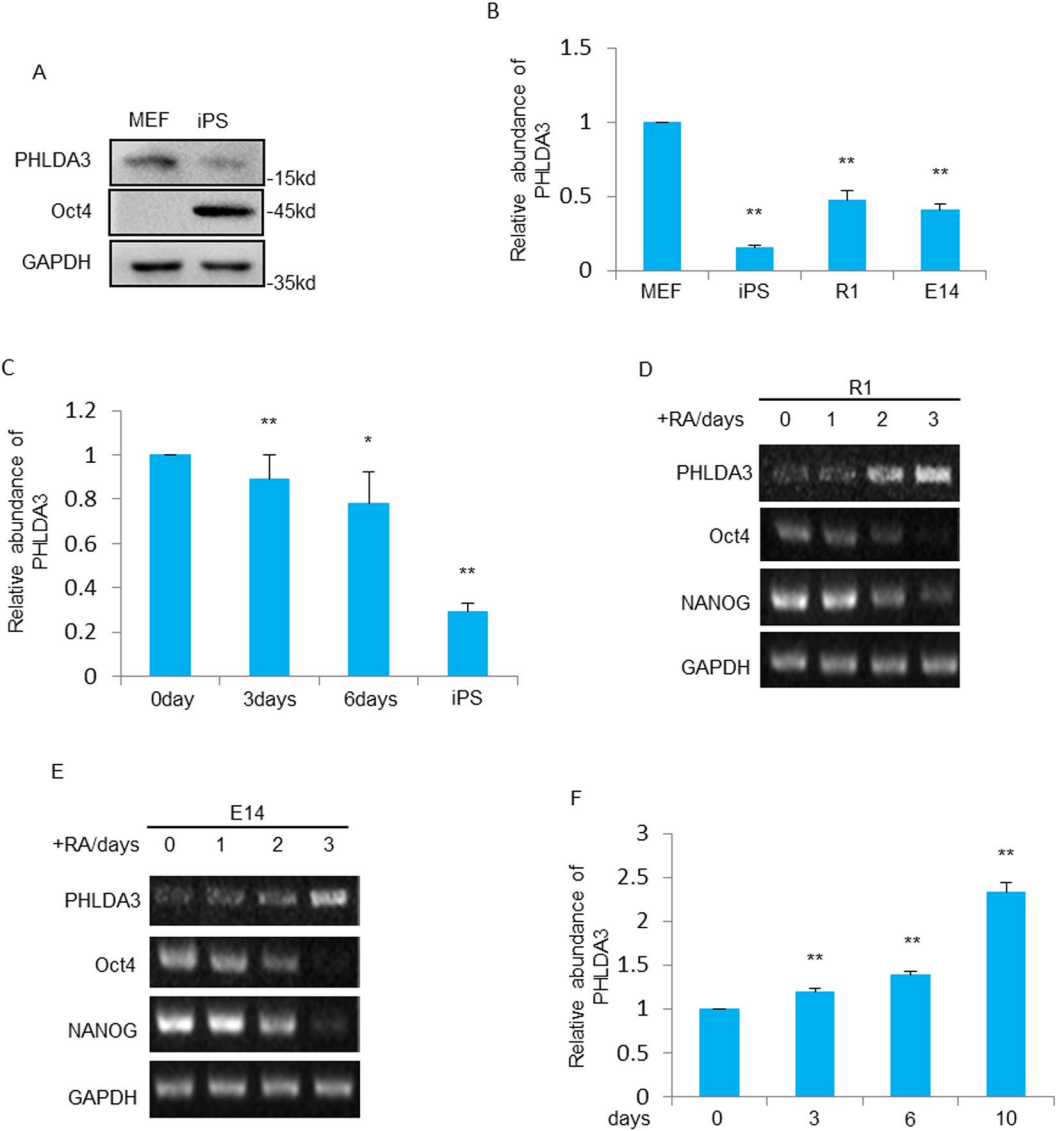
PHLDA3 expression during IPSCs generation and stem cell differentiation. (**A**) PHLDA3 and Oct4 expression in MEF and iPSCs were evaluated by western blot analysis. (**B**) PHLDA3 expression in MEF, iPSCs, R1 and E14 were detected by qRT-PCR analysis. (**C**) PHLDA3 expression in MEF cells infected with retroviruses expressing OSKM for indicated days and iPSCs were analyzed by qRT-PCR assays. (**D**) R1 and E14 (**E**) cells were treated with 0.5 uM of Retinol Acid for indicated days and then subjected to RT-PCR analysis, Oct4 and NANOG were used as controls. (**F**) EB formation was performed using hanging drop method. Cells were collected at indicated times and PHLDA3 expression was analyzed by qRT-PCR.

P53 has been proven to be a blockage of induced pluripotent stem cell generation^16^. However, it remains unknown if PHLDA3, a direct target gene of p53, is involved in this regulation of reprogramming. To address this, we first compared expression levels of PHLDA3 between MEFs (mouse embryonic fibroblasts) and stem cells. PHLDA3 protein was expressed at a lower level in iPSCs than in MEF cells (Fig. 1A). Moreover, PHLDA3 mRNA levels were also higher in MEF cells compared to that in iPSCs and 2 stem cell lines including E14 and R1 (Fig. 1B). We also found that the expression of PHLDA3 was gradually decreased during the process of iPSCs generation (Fig. 1C). Furthermore, in response to retinoic acid (RA)-induced embryonic stem cell differentiation and EB (embryoid body) formation, the expression levels of PHLDA3 were shown to be up-regulated (Fig. 1D,E and F). These data shows that expression levels of PHLDA3 are positively correlated with the differentiation state of stem cells.

### PHLDA3 is a barrier to somatic cell reprogramming

**Figure 2:**
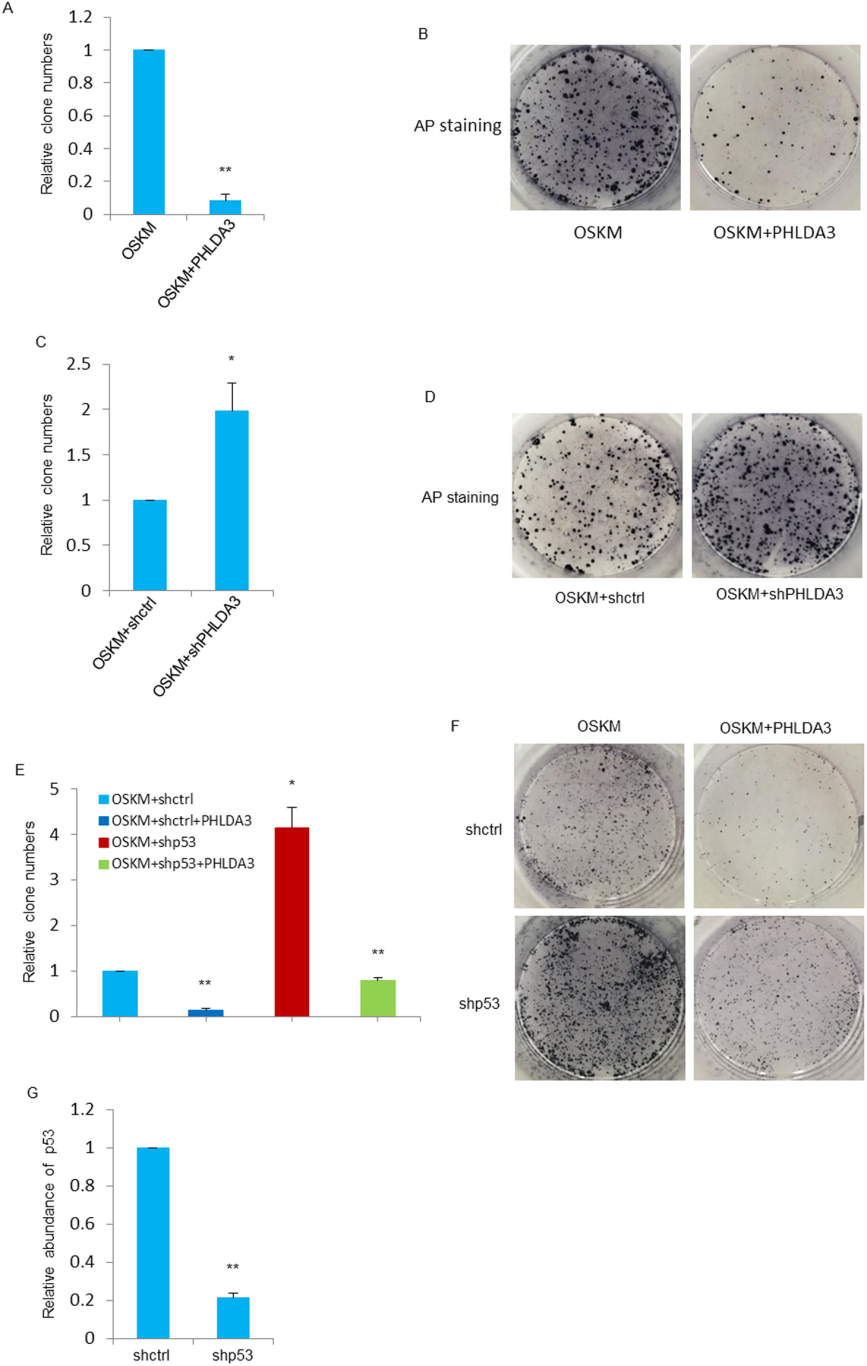
PHLDA3 impedes iPSCs generation. (**A**,**B**) MEF cells expressing OSKM were infected with either pMXs-ctrl or pMXs-PHLDA3 for 2 days and cultured in fresh DMEM for 4 days. GFP positive iPS clones were calculated under fluorescent microscope (**A**). 10 days after infection, iPS clones were transferred onto new feeder cells and maintained in ES medium for another 5 days, then iPS clones were subjected to Alkaline Phosphatase (AP) staining (**B**). (**C**,**D**) MEF cells were infected with OSKM plus either control shRNA or PHLDA3 shRNA and maintained as described in Fig. 2A, iPS clones were counted (**C**) and subjected to AP staining (**D**). (**E**,**F**) MEF cells expressing OSKM were infected with pMXs-PHLDA3, shRNA against p53 or pMXs-PHLDA3 plus shRNA against p53 as indicated. GFP positive iPS clones were calculated (**E**) and subjected to AP staining (**F**). (**G**) p53 knock-down efficiency was evaluated by qRT-PCR analysis.

We next evaluated the effect of PHLDA3 on iPSCs generation. We introduced retroviruses expressing exogenous Oct4, Sox2, Klf4 and c-Myc (OSKM) with or without PHLDA3 into MEF cells. As shown in Fig. 2A and B, overexpression of PHLDA3 with OSKM resulted in an approximately 10-fold decrease in the GFP-positive colonies in reprogramming compared with transduction of OSKM alone. In contrast, knock-down of PHLDA3 increased iPSCs generation efficiency by more than 2 fold (Fig. 2C and D, Supplementary Figures S1 and S7). To validate that iPSCs generated in these experiments are indeed pluripotent, GFP-positive colonies (Supplementary Figure S2) were analyzed for markers of pluripotency with both immunofluorescence (Supplementary Figure S3) and semi-quantitative RT-PCR (Supplementary Figure S4). Taken together, these results demonstrate that depletion of PHLDA3 enhances reprogramming efficiency while overexpression of this gene greatly inhibits iPSCs generation, suggesting that PHLDA3 acts as a barrier to pluripotent reprogramming.

To investigate the relationship between PHLDA3 and P53 during reprogramming, we introduced retroviral-expressing PHLDA3 into MEFs in which P53 was knocked-down. As shown in Fig. 2E–G and Supplementary Figure S6, the elevation of reprogramming efficiency caused by p53 knock-down was reversed by PHLDA3 overexpression. This result shows that the role of inhibiting iPSCs generation by p53 was played, at least partially, through PHLDA3.

### Oct4 transcriptionally inhibits PHLDA3 expression

**Figure 3:**
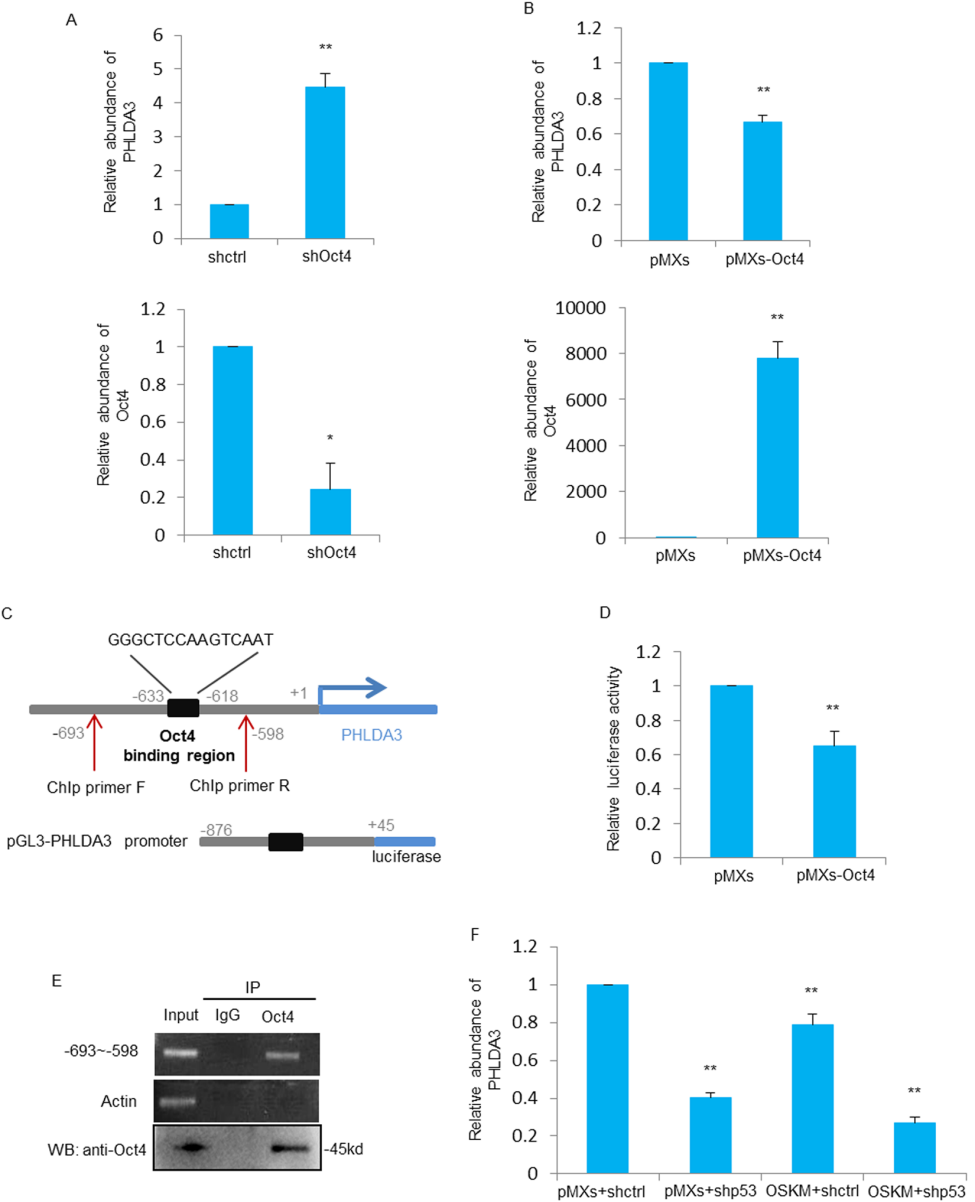
Oct4 transcriptionally inhibits PHLDA3 expression. (**A**) R1 cells were infected with control shRNA or Oct4 shRNA. Oct4 and PHLDA3 expression was detected by qRT-PCR analysis. (**B**) MEF cells were infected with retroviruses expressing control (pMXs) or pMXs- Oct4. Oct4 and PHLDA3 expression was detected by qRT-PCR analysis. (**C**) Schematic illustrations of the putative Oct4 binding region upstream of the PHLDA3 transcriptional start site and the pGL3-basic based PHLDA3 promoter reporter constructs and primers used for ChIp assay. (**D**) B16 cells co-transfected with the reporter constructs, renilla and Oct4 expression plasmids. Twenty-four hours later, cell lysates were subjected to luciferase analysis. (**E**) Genomic DNA from R1 cells was subjected to ChIP assay using Oct4 antibody, and the −693~−598 region of PHLDA3 gene was detected by RT-PCR analysis. Actin was used as a negative control. (**F**) MEF cells expressing pMXs or OSKM were separately infected with either shRNA control or shRNA-p53 as indicated. Six days later, PHLDA3 expression was evaluated by qRT-PCR analysis.

The data above showed that PHLDA3 is down-regulated during iPSCs generation process. To find out which reprogramming gene is responsible for the down-regulation of PHLDA3, several transcription factors which were predicted to possess potential binding sites on PHLDA3 promoter region were selected, including Oct4, Sox2, Esrrb, Tcfap4 and Tcfcp2l1. After knocking down all these genes in R1 cells, Oct4 was found to be the only transcriptional factor that caused PHLDA3 to be up-regulated. Knock-down of Oct4 in R1 cells increased PHLDA3 expression level (Fig. 3A), while overexpression of Oct4 in MEF cells inhibited PHLDA3 expression (Fig. 3B). Further analysis of the PHLDA3 gene promoter had identified a putative Oct4-binding region, which is 618–633 bp upstream of the transcriptional start site (Fig. 3C). Luciferase report assay showed that transfection of Oct4 significantly inhibited the transcriptional activity of the luciferase reporter containing the Oct4-binding region (Fig. 3D). Moreover, Chromatin immunoprecipitation (ChIP) assay showed that Oct4 indeed binds to the demonstrated promoter region of PHLDA3 (Fig. 3E). Taken together, it is proven that Oct4 transcriptionally inhibits PHLDA3 expression. Since p53 also regulates PHLDA3 expression (Supplementary Figure S5), we thought to examine whether these two transcriptional regulators showed additive effect on PHLDA3 expression during the process of iPSCs induction. As shown in Fig. 3f, loss of p53 resulted in a reduced PHLDA3 expression, and further exogenous Oct4 expression led to more down-regulation of PHLDA3 during iPSCs reprogramming, suggesting that these two genes may participate in the regulation of PHLDA3 simultaneously during somatic cell reprogramming.

### PHLDA3 interferes iPSCs generation through activating GSK3β

**Figure 4:**
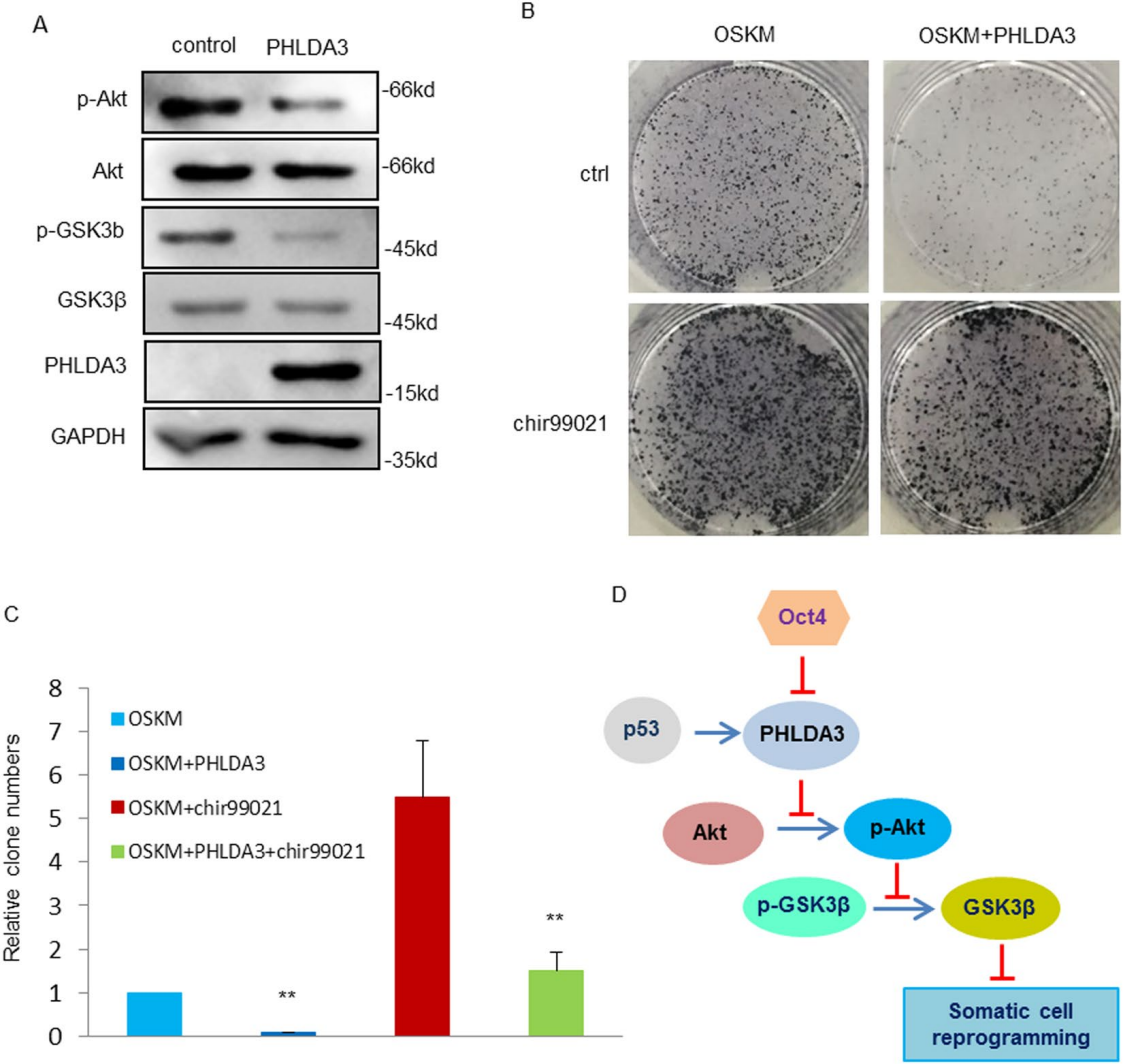
PHLDA3 inhibits iPSCs generation through activating GSK3β. (**A**) MEF cells expressing OSKM were infected with either pMXs or pMXs-PHLDA3 for 4 days, then cell lysates were analyzed with the indicated antibodies. (**B**,**C**) MEF cells expressing OSKM were infected with either pMXs or pMXs-PHLDA3, then MEF cells were induced for iPSCs with and without 3 uM chir99021. GFP positive clones were calculated at 10 days (**C**) and then transferred onto new feeder cells for another 5 days before subjected to AP staining (**B**). (**D**) A schematic illustration of the proposed model depicting a PHLDA3-mediate pathway to impede somatic cell reprogramming.

In order to further reveal the molecular mechanism underlying the effect of PHLDA3 on the generation of iPSCs, we examined its downstream signaling pathways during iPSCs reprogramming. It was reported that PHLDA3 decreases Akt kinase activity through inhibiting Akt phosphorylation, and in turn Akt inactivates GSK3β by phosphorylating GSK3β^14, 17^. To determine whether these pathways were also inhibited by PHLDA3 during reprogramming, the phosphorylation levels of Akt and GSK3β during the process of iPSCs generation were detected. MEF cells were infected with OSKM plus either control or PHLDA3 for OSKM-mediated somatic cell reprogramming, and as shown in Fig. 4A, PHLDA3 overexpression indeed resulted in the down-regulation of Akt and GSK3β phosphorylation. This result shows that overexpression of PHLDA3 activates GSK3β, a kinase that blocks iPSCs generation.

We next examined whether the inhibition of iPSCs generation by PHLDA3 is dependent on GSK3β activation. As shown in Fig. 4B and C, reprogramming efficiency was inhibited by PHLDA3 overexpression, and this effect was rescued by addition of GSK3β inhibitor CHIR99021, which indicates that function of PHLDA3 on reprogramming is at least partially through activating GSK3β.

To investigate the effect of Akt on iPSC generation, we treated cells with an Akt inhibitor, MK2206 during the iPSCs induction. As shown in Supplementary Figure S8, suppression of Akt led to a decrease in p- GSK3β and reprogramming efficiency. Furthermore, increased reprogramming efficiency caused by PHLDA3 knockdown can be reversed by addition of MK2206. Taken together, PHLDA3 indeed affects reprogramming through Akt-GSK3β pathway.

Collectively, our experiments have shown that PHLDA3 is a novel participant in the regulatory network in somatic cell reprogramming. It is suppressed by Oct4, the key booster of somatic cell reprogramming, and up-regulated by p53, which is known to be a barrier to iPSCs generation. During somatic cell reprogramming, PHLDA3 results in a decrease in the phosphorylation of both Akt and GSK3β, suggesting that PHLDA3 activates Akt-GSK3β pathway, which is also a barrier to iPSCs generation (Fig. 4D).

## Discussion

In this study, we demonstrate that PHLDA3 is a novel repressor of somatic cell reprogramming. It is transcriptionally up-regulated by p53, a well-known blockage of somatic cell reprogramming^14, 16^, and is also transcriptionally repressed by Oct4, one of the key transcriptional regulators contributing to iPSCs generation. PHLDA3 activates Akt-GSK3β pathway during the reprogramming process, resulting in the decrease of iPSCs generating efficiency. These studies show that PHLDA3 is an important part of the signaling network regulating somatic cell reprogramming.

The method of iPSCs induction utilizing the Yamanaka factors has been widely used throughout the world. Yet the reprogramming efficiency by OSKM induction is extremely low, suggesting that various barriers exist during the reprogramming process. Finding out and removing these barriers will not only provide new methods to improve iPSCs induction efficiency, but also shed new lights on the mechanism of somatic cell reprogramming^3, 4, 18^. Over the past years, many genes have been found to affect iPSCs generation, yet for the vast majority of those genes, the molecular mechanism underlying this regulation in this process remains largely uncharacterized. PHLDA3 is previously known as a tumor repressor^19, 20^, in this study, we found that this gene also participates in somatic cell reprogramming.

As an important factor to suppress tumorigenesis, p53 has been reported as a repressor of somatic cell reprogramming and self-renewal of stem cells^21^. Knock-down of p53 has been adopted as basis to improve the efficiency of iPSCs generation^16^. p53 is able to induce cell senescence and apoptosis, which was suggested to be responsible for its function in iPSCs generation and stem cell self-renewal^21^. It is worth noting that p53-regulated genes or noncoding RNAs, such as p21^16^, MDM2^22^, miR-199a-3p^23^ and lncRNA-p21^24^, have also been found to participate in the regulation of the reprogramming process. In this study, another p53 target gene, PHLDA3, is added to the list. It expands our knowledge of the network of p53 in somatic cell reprogramming.

Interestingly, although iPSCs and ESCs share lots of common features, we did not find that PHLDA3 is important to ES cell differentiation. After inducing exogenous PHLDA3 into E14 cell lines, no significant morphology change of the ES clones was observed (data not shown). This suggests that in ESCs, PHLDA3 may adopt a distinct downstream pathway which is not applicable to iPSCs generation. Further investigation is needed to elucidate the detailed molecular mechanisms of this pathway.

## Materials and Methods

### Cell culture and iPSCs induction

MEF and 293 T cells were maintained in Dulbecco’s modified Eagle’s medium (DMEM) supplemented with 10% fetal bovine serum (FBS). Mouse embryonic stem cell lines R1 and E14 were maintained in DMEM supplemented with 10% FBS, 10% KSR, 2 mM L-Glutamine, 100 μM Non Essential Amino Acids (NEAA), 0.1 mM ß-Mercaptoethanol, 1 mM sodium pyruvate (Invitrogen) and 1000 U/ml LIF (Millipore).

Retrovirus used for iPSCs generation was obtained using plat-E packaging cells. pMXs plasmids containing mouse Oct4, Sox2, Klf4, or c-Myc (Addgene) were transfected into plat-E cells with lipofectamin. 12 hours after transfection, cells were cultured with fresh medium for additional 36 hours. The culture medium containing retrovirus particles was centrifuged at 1,000 g for 5 minutes, and the supernatant was used for infection.

Oct4-EGFP MEF cells isolated from E13.5 Embryos and seeded at a density of 3 × 10^4^ cells per well in six-well plate for 24 hours before infected with indicated retroviruses supplemented with 8 ug/ml polybrene. Fresh medium were changed 24 hours after virus infection.

Cells were transferred onto feeder cells 4~6 days after infection and cultured in DEME supplemented with 15% KSR, 2 mM L-Glutamine, 100 μMNon Essential Amino Acids (NEAA), 0.1 mM ß-Mercaptoethanol, 1 mM sodium pyruvate (Invitrogen) and 1000 U/ml LIF (Millipore).

### Lentivirus package and RNA interference

Lentivirus packaging was performed using a protocol described previously^25^. To generate lentivirus expressing shRNAs, HEK 293 T cells were transfected with shRNAs (cloned in PLKO.1), gag/pol, rev and VSVG plasmids with the ratio of 2:2:2:1. Twenty-four hours after transfection, cells were cultured with fresh DMEM for an additional 24 hours. The culture medium containing lentivirus particles was centrifuged at 1,000 g for 5 minutes and the supernatant were used for infection. Followed are sequences used to knockdown PHLDA3, Oct4 and p53: PHLDA3: 5′-GCTATCAACATGAGGAAGATA-3′ and 5′-CCCTTTCTTTGCACACTTCTT-3′; Oct4: 5′-GCCGACAACAATGAGAACCTT-3′ and 5′-CCTACAGCAGATCACTCACAT-3′; p53: 5′-CGGCGCACAGAGGAAGAGAAT-3′ and 5′-TCAGACCTATGGAAACTACTT-3′.

### Western Blot Analysis

Western Blot analysis was performed as described previously^26^. The following antibodies were obtained from the indicated sources: GAPDH and Oct4 (Santa Cruz), Sox2 and Nanog (Millipore), PHLDA3 (Abcam).

### qRT-PCR and RT-PCR

Total RNA was isolated using Trizol (Invitrogen). One microgram of total RNA was used to synthesize cDNA using PrimeScript TM RT reagent kit (Takara) according to the manufacturer’s instruction. qRT-PCR was performed using SYBR premix EX Taq (TaKaRa) and ROX and analyzed with Stratagene Mx3000p (Agilent). RT-PCR was performed using Taq Mix (TIANGEN). The following primers were used in this study:

Oct4: Forward: 5′-CTGTAGGGAGGGCTTCGGGCACTT-3′ and

Reverse: 5′-CTGAGGGCCAGGCAGGAGCACGAG-3′;

Nanog: Forward: 5′-AGGGTCTGCTACTGAGATGCTCTG-3′ and

Reverse: 5′-CAACCACTGGTTTTTCTGCCACCG-3′;

PHLDA3: Forward: 5′-CCGTGGAGTGCGTAGAGAG-3′ and

Reverse: 5′-TCGGTCACTAGCGTGAAGTAG-3′;

Esrrb: Forward: 5′-TGGCAGGCAAGGATGACAGA-3′ and

Reverse: 5′-TTTACATGAGGGCCGTGGGA-3′;

Klf5: Forward: 5′-CCGGAGACGATCTGAAACAC-3′ and

Reverse: 5′-CAGATACTTCTCCATTTCACATCTTG-3′;

Eras: Forward: 5′-GCCCCTCATCAGACTGCTAC-3′ and

Reverse: 5′-GCAGCTCAAGGAAGAGGTGT-3′;

fgf4: Forward: 5′-CTACTGCAACGTGGGCATCG and

Reverse: 5′-CGCTGCACCGGAGAGAGC;

Dppa4: Forward: 5′-AAGGGCTTTCCCAGAACAAT and

Reverse: 5′-TCCAGAGGAACTGTCACCTCA-3′.

### Alkaline phosphatase staining

Emerging iPS cells were subjected to alkaline phosphatase (AP) staining by using the Alkaline Phosphatase Staining Kit (Beyotime) according to the manufacturer’s protocol.

### Immunofluorescence

IPS cells were seeded on 0.1% gelatin-coated plate. Three days after seeding, cells were fixed with 4% paraformaldehyde, permeabilized with 0.1% Triton-X-100 in phosphate buffered saline (PBS), and blocked with 1% Bovine serum albumin in PBS. Cells were then incubated at room temperature with anti-Oct4 (Santa Cruz) or anti-Ssea1 (Santa Cruz) antibody for 2 hours and with rhodamine-conjugated anti-rabbit IgG secondary antibody for an additional 1 hour. The cells were washed twice with PBS containing 0.1% Tween20 and stained with Hoechst 33342 (Sigma). Images were captured with an Olympus fluorescence microscope.

### Embryoid bodies (EBs) Formation

Hanging drops method was used to obtain embryoid bodies. Briefly, R1 cells were dissociated into single cells at the density of 200–300 cell/ul and cultured in hanging drops (20 ul/drop) for 3 days before seeded on attached plates for further culturing.

### ChIP Assay

ChIP Assay was performed using ChIP Assay Kit (Beyotime) according to the manufacturer’s instruction. Briefly, cells were cross-linked with Formaldehyde, subjected to ultrasonic disruption until DNA was fragmented into 200–1000 bp, and then incubated with beads coated with antibodies against Oct-4 in ChIP dilution buffer. DNA fragments that bind with Oct-4 were eluted with Elution buffer and subjected to PCR analysis.

### Statistical Analysis

Statistical analysis was carried out using Microsoft Excel software to assess differences between varying groups. Statistical significance was analyzed by Student’s t test and expressed as a p value. p values lower than 0.05 were considered to be statistical significance. * and ** indicate p < 0.05 and p < 0.01, respectively.

## Electronic supplementary material

Supplementary Figures

## References

[1] Wernig M (2007). *In vitro* reprogramming of fibroblasts into a pluripotent ES-cell-like state. Nature.

[2] Okita K, Ichisaka T, Yamanaka S (2007). Generation of germline-competent induced pluripotent stem cells. Nature.

[3] Takahashi K, Yamanaka S (2006). Induction of Pluripotent Stem Cells from Mouse Embryonic and Adult Fibroblast Cultures by Defined Factors. Cell.

[4] Takahashi K (2007). Induction of Pluripotent Stem Cells from Adult Human Fibroblasts by Defined Factors. Cell.

[5] Maherali N (2007). Directly reprogrammed fibroblasts show global epigenetic remodeling and widespread tissue contribution. Cell Stem Cell.

[6] Boyer LA (2005). Core Transcriptional Regulatory Circuitry in Human Embryonic Stem Cells. Cell.

[7] Matsuda T (1999). STAT3 activation is sufficient to maintain an undifferentiated state of mouse embryonic stem cells. The EMBO Journal.

[8] Williams RL (1988). Myeloid leukaemia inhibitory factor maintains the developmental potential of embryonic stem cells. Nature.

[9] Ying Q-L, Nichols J, Chambers I, Smith A (2003). BMP Induction of Id Proteins Suppresses Differentiation and Sustains Embryonic Stem Cell Self-Renewal in Collaboration with STAT3. Cell.

[10] Ying Q-L (2008). The ground state of embryonic stem cell self-renewal. Nature.

[11] Chen X (2008). Integration of external signaling pathways with the core transcriptional network in embryonic stem cells. Cell.

[12] Kim J, Chu J, Shen X, Wang J, Orkin SH (2008). An Extended Transcriptional Network for Pluripotency of Embryonic Stem Cells. Cell.

[13] Loh YH (2006). The Oct4 and Nanog transcription network regulates pluripotency in mouse embryonic stem cells. Nature genetics.

[14] Kawase T (2009). PH Domain-Only Protein PHLDA3 Is a p53-Regulated Repressor of Akt. Cell.

[15] Ohki R (2014). PHLDA3 is a novel tumor suppressor of pancreatic neuroendocrine tumors. Proceedings of the National Academy of Sciences of the United States of America.

[16] Hong H (2009). Suppression of induced pluripotent stem cell generation by the p53-p21 pathway. Nature.

[17] Alao, J. P. The regulation of cyclin D1 degradation: roles in cancer development and the potential for therapeutic invention. *Mol Cancer* **6**, doi: 10.1186/1476-4598-6-24 (2007). 10.1186/1476-4598-6-24 PMC1851974 17407548

[18] Lu X, Zhao T (2013). Clinical therapy using iPSCs: hopes and challenges. Genomics Proteomics Bioinformatics.

[19] Kawase T (2009). PH Domain-Only Protein PHLDA3 Is a p53-Regulated Repressor of Akt. Cell.

[20] Ohki R (2014). PHLDA3 is a novel tumor suppressor of pancreatic neuroendocrine tumors. Proceedings of the National Academy of Sciences.

[21] Puzio-Kuter AM, Levine AJ (2009). Stem cell biology meets p53. Nat Biotechnol.

[22] Wienken M (2016). MDM2 Associates with Polycomb Repressor Complex 2 and Enhances Stemness-Promoting Chromatin Modifications Independent of p53. Molecular Cell.

[23] Wang J (2012). p53-facilitated miR-199a-3p regulates somatic cell reprogramming. Stem Cells.

[24] Bao X (2015). The p53-induced lincRNA-p21 derails somatic cell reprogramming by sustaining H3K9me3 and CpG methylation at pluripotency gene promoters. Cell Res.

[25] Li, Q. *et al*. C23 promotes tumorigenesis via suppressing p53 activity. *Oncotarget* (2016). 10.18632/oncotarget.11071 PMC5295430 27506938

[26] Mei Y (2007). Noxa/Mcl-1 Balance Regulates Susceptibility of Cells to Camptothecin-Induced Apoptosis. Neoplasia.

