# Supplementary material for: PHLDA3 impedes somatic cell reprogramming by activating Akt-GSK3β pathway
Source: Sci Rep. 2017 Jun 6;7:2832. doi: 10.1038/s41598-017-02982-9 (PMC5460190; doi:10.1038/s41598-017-02982-9)
Supplement: Supplementary file 1 — Supplementary Figures [file 41598_2017_2982_MOESM1_ESM.pdf]

Supplemental Information

PHLDA3 impedes somatic cell reprogramming by activating Akt-GSK3β pathway

Mengran Qiao, Mian Wu, Ronghua Shi ,and Wanglai Hu

S1

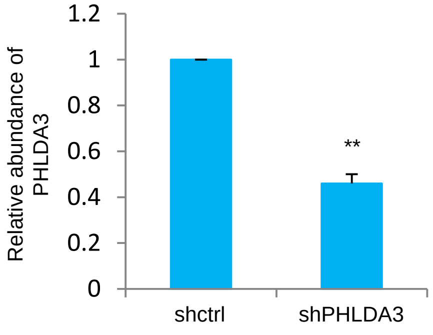
1.2
1
0.8
0.6
0.4
0.2
0
Relative abundance of PHLDA3
**
shctrl
shPHLDA3

S2

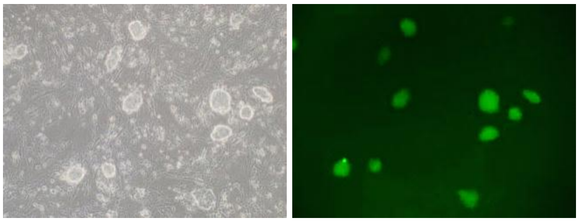

Bright field   Fluorescent

S3

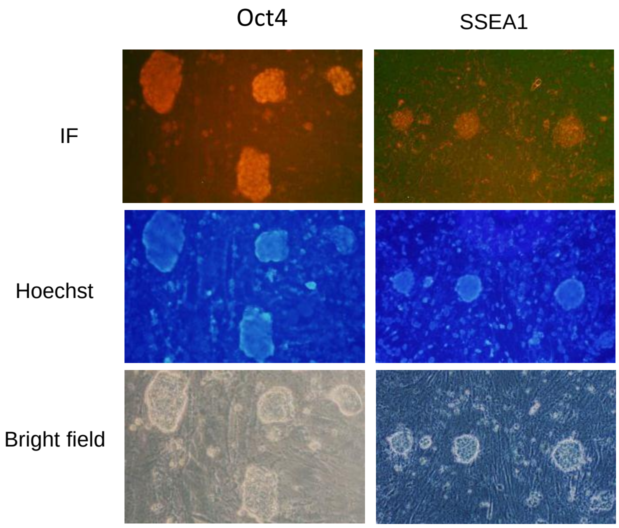

S4

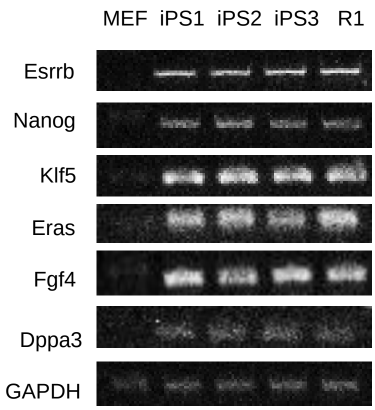

S5

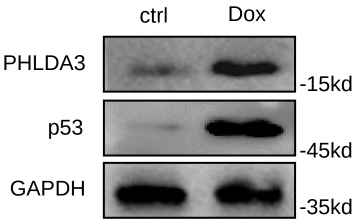

S6

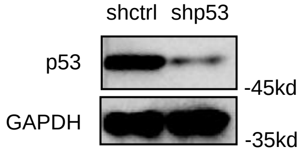

S7

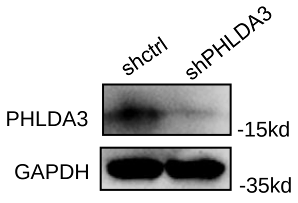

S8

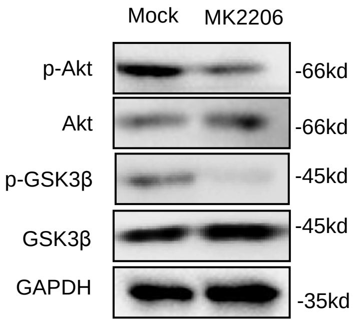

Mock
MK2206
p-Akt
Akt
p-GSK3β
GSK3β
GAPDH
-66kd
-66kd
-45kd
-45kd
-35kd

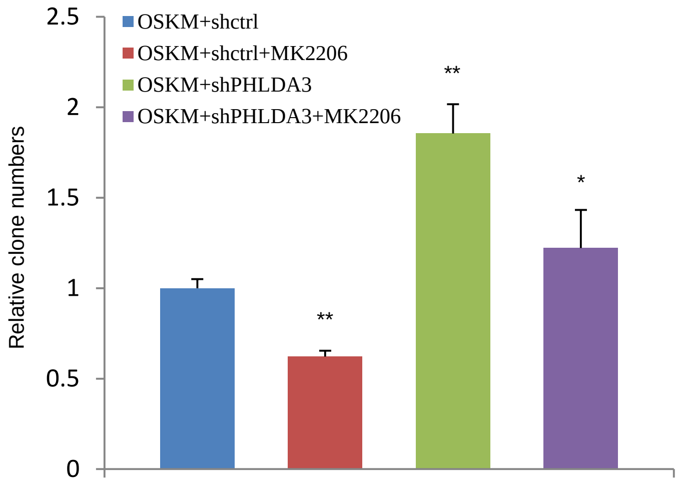

OSKM+shctrl
OSKM+shctrl+MK2206
OSKM+shPHLDA3
OSKM+shPHLDA3+MK2206
Relative clone numbers
2.5
2
1.5
1
0.5
0
**
**
*

Supplemental Figure Legends

Figure S1. PHLDA3 knock-down efficiency was examined by qRT-PCR analysis.

Figure S2. iPSCs were observed under fluorescent microscopes.

Figure S3. Oct4 and SSEA1 expression in iPSCs were tested using immune-fluoresce.

Figure S4. Pluripotent marker genes expression in iPSCs were analyzed by RT-PCR.

Figure S5. MEF cells were treated with and without 0.2uM doxorubicin for 10 h and then subjected to western blot analysis with indicated antibodies.

Figure S6. PHLDA3 knock-down efficiency was examined by western blot analysis.

Figure S7. Knock-down efficiency was examined by western blotting.

Figure S8. MEF cells were infected with OSKM and either shctrl or shPHLDA3 for 2 days, and then treated with or without MK2206 during somatic cell reprogramming, cells were collected for western blot analysis, and GFP positive clones were calculated under fluorescent microscope.
